# Exogenous aspartic acid alleviates salt stress-induced decline in growth by enhancing antioxidants and compatible solutes while reducing reactive oxygen species in wheat

**DOI:** 10.3389/fpls.2022.987641

**Published:** 2022-10-17

**Authors:** Mervat Sh Sadak, Agnieszka Sekara, Ibrahim Al-ashkar, Muhammad Habib-ur-Rahman, Milan Skalicky, Marian Brestic, Ashwani Kumar, Ayman El Sabagh, Magdi T. Abdelhamid

**Affiliations:** ^1^ Botany Department, National Research Centre, Cairo, Egypt; ^2^ Department of Horticulture, The University of Agriculture in Krakow, Kraków, Poland; ^3^ Department of Plant Production, College of Food and Agriculture, King Saud University, Riyadh, Saudi Arabia; ^4^ Institute of Crop Science and Resource Conservation (INRES), University of Bonn, Crop Science, Bonn, Germany; ^5^ Department of Botany and Plant Physiology, Faculty of Agrobiology, Food, and Natural Resources, Czech University of Life Sciences Prague, Prague, Czechia; ^6^ Department of Plant Physiology, Slovak University of Agriculture, Nitra, Slovakia; ^7^ Metagenomics and Secretomics Research Laboratory, Department of Botany, Dr. HarisinghGour Central University, Sagar, MP, India; ^8^ Department of Agronomy, Faculty of Agriculture, Kafrelsheikh University, Kafrelsheikh, Egypt

**Keywords:** Antioxidant enzymes, aspartic acid, hydrogen peroxide, lipid peroxidation, osmoprotectants, salinity stress, Triticum aestivum

## Abstract

Salinity is the primary environmental stress that adversely affects plants’ growth and productivity in many areas of the world. Published research validated the role of aspartic acid in improving plant tolerance against salinity stress. Therefore, in the present work, factorial pot trials in a completely randomized design were conducted to examine the potential role of exogenous application of aspartic acid (Asp) in increasing the tolerance of wheat (*Triticum aestivum* L.) plants against salt stress. Wheat plants were sown with different levels of salinity (0, 30, or 60 mM NaCl) and treated with three levels of exogenous application of foliar spray of aspartic acid (Asp) (0, 0.4, 0.6, or 0.8 mM). Results of the study indicated that salinity stress decreased growth attributes like shoot length, leaf area, and shoot biomass along with photosynthesis pigments and endogenous indole acetic acid. NaCl stress reduced the total content of carbohydrates, flavonoid, beta carotene, lycopene, and free radical scavenging activity (DPPH%). However, Asp application enhanced photosynthetic pigments and endogenous indole acetic acid, consequently improving plant leaf area, leading to higher biomass dry weight either under salt-stressed or non-stressed plants. Exogenous application of Asp, up-regulate the antioxidant system *viz*. antioxidant enzymes (superoxide dismutase, peroxidase, catalase, and nitrate reductase), and non-enzymatic antioxidants (ascorbate, glutathione, total phenolic content, total flavonoid content, beta carotene, lycopene) contents resulted in declined in reactive oxygen species (ROS). The decreased ROS in Asp-treated plants resulted in reduced hydrogen peroxide, lipid peroxidation (MDA), and aldehyde under salt or non-salt stress conditions. Furthermore, Asp foliar application increased compatible solute accumulation (amino acids, proline, total soluble sugar, and total carbohydrates) and increased radical scavenging activity of DPPH and enzymatic ABTS. Results revealed that the quadratic regression model explained 100% of the shoot dry weight (SDW) yield variation. With an increase in Asp application level by 1.0 mM, the SDW was projected to upsurge through 956 mg/plant. In the quadratic curve model, if Asp is applied at a level of 0.95 mM, the SDW is probably 2.13 g plant^-1^. This study concluded that the exogenous application of aspartic acid mitigated the adverse effect of salt stress damage on wheat plants and provided economic benefits.

## 1 Introduction

Salinity is one of the highly harmful environmental stress, which decreases growth as well as yield of crops in various soil types of the world in rainfed and semi-rainfed areas. The adverse effect of salinity on plant growth is due to the unavailability of available water to plants, which causes osmotic stress and impairs useful functions (Hasanuzzaman and Fujita, 2022). Salinity causes various adverse effects on plant morphology, physiology, and metabolic processes, resulting from disrupted redox homeostasis of the plant (Alharbi et al., 2021; Rehman et al., 2021; Ahmed et al., 2022). The overproduction of reactive oxygen species (ROS), damages cellular membranes *via* inducing lipid peroxidation, followed by disturbances in DNA and RNA replication and enzymatic protein functioning (Mittova et al., 2002). To overcome these adverse effects of ROS, plant activates acclimation mechanisms such as increasing the levels of osmoprotectants and by enhancing the antioxidant system (Bekheta et al., 2009; Rady et al., 2016; Singhal et al. (2021). The formation and accretion of osmoprotectants or compatible osmolytes are common mechanisms by which plants can tolerate abiotic stresses (Rady et al., 2015). The antioxidant system comprises of a nonenzymatic antioxidant system e.g. ascorbic acid, glutathione, and tocopherols (Sharma and Dubey, 2007), as well as the antioxidative enzymatic system such as superoxide dismutase (SOD), catalase (CAT), peroxidase (POX), and nitrate reductase (NR) (El-Lethy et al., 2013).

The wheat crop is among the oldest and utmost important grain crops worldwide (*Triticum aestivum* L.). Since the green revolution, crops recorded a threefold increase in average yields, approaching the potential yield threshold. With a rapidly rising population, such an increase in average crop yields is inadequate to meet food security goals in developing countries, where the yield gap is significant. Egypt is a leading wheat importer, as local wheat production is insufficient to meet wheat demand. In Egypt, wheat is ranked 2^nd^ (after rice) in terms of dietary intake (Abd El-Rheem et al., 2015). Wheat provides approximately one-fifth of the calories in the human diet (Hussein et al., 2015). Wheat is a moderately salt-tolerant crop (Maas and Hoffman, 1977; Mujeeb-Kazi et al., 2019; Saddiq et al., 2021), furthermore, efforts have been made to incorporate all three types of tolerance, i.e., tissue and osmotic tolerance and ion exclusion. In the cradle of agriculture (Nile Valley) wheat yield can be increased, under saline and normal conditions, by developing new high-yielding varieties and incorporating genetic and agronomic improvements to extend wheat production from core areas to marginal lands (Orabi and Sadak, 2015).

Improvement of plant productivity under different environmental stresses is essential. This can be attained by generating the latest adaptable varieties with higher yield potential and applying appropriate agricultural practices (Abdelhamid et al., 2003; Abdelhamid et al., 2011; El Sabagh et al., 2021). These practices includes applications of biofertilizers, mineral fertilizer as a source of phosphorus and potassium (Dawood et al., 2014; Mohamed et al., 2021), inoculation of an exopolysaccharides (EPS) producing bacterial strains (Awad et al., 2012), as well as using various naturally occurring compounds as exogenous plant treatment such as vitamins, antioxidants, amino acids, etc. The application of amino acids is one of these approaches used to increase plant productivity in sub-optimal conditions (Abd Elhamid et al., 2016). The amino acids are organic nitrogenous compounds involved in synthesizing proteins produced by polymerizing amino acids. They are known as growth factors of higher plants as constituents of proteins, including enzymatic proteins and non-proteinic nitrogenous compounds, i.e., pigments, vitamins, coenzymes, purine, and pyrimidine derivates (Davies, 1982). Furthermore, the application of amino acids can lessen the applied quantities of inorganic fertilizers as well as can cause the improvement in the qualitative traits of plants through enhancement of the macro and micro nutrients uptake as well as increase nutrients use efficiency for the gain maximum attainable yield (Meijer, 2003). Amino acids, as biological stimulants, have been proven in recent research to increase physiological features, dramatically improve salt tolerance, and reduce the effects of salt stress on plant growth and development (Xu et al., 2018).

Aspartic acid (Asp) is α-amino acid necessary for protein production. Aspartate, also known as aspartic acid, is a basic amino acid that is used in the production of other amino acids, nucleotides, organic acids in the tricarboxylic acid (TCA) cycle, sugars in glycolysis, and hormones, all of which are important for plant development and stress resistance (Han et al., 2021). Furthermore, Asp assimilates inorganic nitrogen, supplying a nitrogen source for plants to synthesize other nitrogen-containing molecules (Buchanan and Bb, 1980). Because Asp plays a vital role in producing other metabolites, the Asp-derived amino acid pathway has sparked a lot of interest in plant nutrition (Jander and Joshi, 2010). Aspartic acid is required for cell proliferation and role in vegetation responses to abiotic stressors (Simon-Sarkadi and Galiba, 1996; Farhangi-Abriz and Ghassemi-Golezani, 2016; Han et al., 2021). The endogenous content of Asp increases or decreases in response to abiotic stress (e.g., cold stress, drought stress, salt stress, and heavy metal stress) depending on the plant species and kind of stress (Naidu et al., 1991; Simon-Sarkadi and Galiba, 1996; Farhangi-Abriz and Ghassemi-Golezani, 2016; Rizwan et al., 2016). Under salt stress, Asp accumulates with other amino acids like proline, which is linked to its involvement in osmotic adjustment and membrane stability (Hayat et al., 2012). Although the underlying metabolic pathways are unknown, exogenous application of Asp can improve plant tolerance to salt (Zhang et al., 2020), heat stress (Lei et al., 2022), and cadmium toxicity (Rizwan et al., 2017b) based on plant growth and physiological responses. In plants, vascular tissues are responsible for transporting wide-ranging types of amino acids with varying concentrations. However, glutamate, aspartate, and their derivates are the most abundant (Tegeder and Masclaux-Daubresse, 2018). In the younger plants of crops, the formation of amino acids is controlled through the metabolic process of glutamate, aspartate, and their derivatives, which are assimilated into other amino acids *via* numerous biochemical processes (Davies, 1982). The influence of the aspartic acid on different parts of plant morphology and physiological processes has encouraged several researchers to apply them to numerous crops. The primary purpose is to control the growth pattern and developmental stages and enhance systemic tolerance against stress-causing agents.

There is not much research out there right now on how aspartic acid can alter the physiological traits of crops under salt stress. To examine the effect of aspartic acid on physiological characteristics of salt exclusion in wheat under salt stress, antioxidant enzymes and non-enzymatic antioxidant contents, and reactive oxygen species (ROS) were measured. In this work, the hypothesis was that with foliar application of aspartic acid in various concentrations, plants of wheat crops display enhanced traits related to growth under stress caused due to salinity. This study will serve as the foundation for the study of the mechanisms by which exogenous substances improve salt tolerance in crops.

## 2 Materials and methods

### 2.1 Experimental procedure

During the winter season of 2016/17, a pot experiment was carried out at the National Research Centre’s greenhouse in Dokki, Cairo, Egypt, to investigate the exogenous effect of foliar Asp treatments on wheat plants irrigated with saline water.

Daily temperatures ranged from 12.3 to 17.0°C throughout the experiment, with an average of 15.4 ± 1.0°C. The average night temperature was 13.1 ± 1.6°C, with minimum and highest temperatures of 8.7 and 16.7°C, respectively, and the average day temperature was 23.1 ± 2.8°C, with minimum and maximum temperatures of 17.8 and 28.4°C, respectively. The daily relative humidity ranged from 22.1 to 59.2%, with a mean of 49.7% ± 9.2%.

Wheat (*Triticum aestivum* L.) var. Misr 1 grains were chosen for uniformity by selecting those of identical size and color. The grains were carefully rinsed with distilled water before being sterilized with a 1% sodium hypochlorite solution for about 2 minutes. Ten uniforms, air-dried wheat grains were sown in a plastic pot (7 litre pot size) with 25.0 cm pot diameter (top), 19.0 cm pot diameter (base), and 20.0 cm pot height at a depth of 30 mm, in approx. 7.0 kg of clay soil. Polyethylene bags are used to cover each pot to prevent water loss through evaporation. The following were the physical and chemical characteristics of the experimental soil: The soil texture was clay-loam, with coarse sand 1.4%, fine sand 31.7%, silt 39.6%, and clay 27.3%, EC_e_ 1.82 dS m^-1^, pH 7.5, organic matter 1.93%, CaCO_3_ 7.88%, and available N, P, and K accounting for 45.6, 7.8 and 415.0 mg kg^-1^, respectively. The soil was blended with yellow sand in a 3:1 ratio to minimize compaction and promote drainage (v:v). The seedlings were trimmed to 5 seedlings per pot ten days after seeding. Three days before cultivation, the mineral fertilizers were mixed with the soil in the following doses: (1) ammonium sulfate (20.5% N) at an 800 kg ha^-1^ dose; (2) super phosphate (15% P_2_O_5_) at a 240 kg ha1 dose; and (3) potassium sulfate (48% K_2_O) at a 120 kg ha^-1^ dose. Before planting, the N, P, and K fertilizers were completely mixed into the soil of each pot. By saturating the soil in the pots with water, draining for 48 hours, and weighing, the soil water field capacity, 0.36, was calculated. The soil water capacity was kept at around 90% of its maximum capacity by weighing the pots and balancing the daily water loss. All agricultural procedures for wheat production were carried out following the Egyptian Ministry of Agriculture and Land Reclamation’s instructions.

The experiment was factorial in a completely randomized design and replicated three times. The experiment comprised two factors. The first factor included three saline water levels namely 0.0, 30, or 60 mmol/L (mM) sodium chloride (NaCl). The second factor involved four levels of Asp *viz*. 0, 0.4, 0.6 or 0.8 mmol/L(mM). Therefore, the experiment consisted of twelve treatments as combinations of 3 salt irrigation regimes (NaCl) and four Asp levels. NaCl and Asp were purchased from Sigma-Aldrich, Saint Louis, MO, USA. After plant emergence, wheat seedlings were thinned ten days after sowing (DAS), and four plants per pot were left. Pots were irrigated with distilled water supplemented with 0.0, 30, or 60 mM NaCl starting from 15 DAS and lasted till the harvesting of wheat plants at 75 DAS. Spraying wheat plants with Asp was done at 42 and 56 DAS, coinciding with stem elongation or jointing stage GS31: First node detectable, and GS39: Flag leaf fully unrolled, ligule just visible, respectively. All spray treatments were completed early morning, before 9:00 a.m., with a hand sprayer at sufficient pressure to keep droplet size small. To obtain proper coverage, plants were sprayed from all sides. The spray volume amount of water consisted of approximately 500 L per ha based on each pot area is 0.049 m2. Plants were sprayed from both sides of the row to achieve adequate coverage.

### 2.2 Measurements

After 75 days, plant samples were taken to assess growth characteristics such as shoot length (cm), leaf area (dm^2^ plant^-1^), and shoot dry weight (g plant^-1^).

After 75 DAS, plant samples were obtained for chemical analysis. The following biochemical parameters were analysed i.e., photosynthetic pigments, indoleacetic acid (IAA), compatible solute accumulation (amino acids; AA, proline; Pro, total soluble sugar; TSS, and total carbohydrates; TC), hydrogen peroxide (H_2_O_2_), lipid peroxidation (MDA), aldehyde (ALD), enzymatic antioxidants (superoxide dismutase; SOD, peroxidase; POX, catalase; CAT, and nitrate reductase; NR), non-enzymatic antioxidants (glutathione (GSH), ascorbate; AsA, total phenolic content (TPC), total flavonoid concentration; TFC, beta carotene; B-Car, lycopene; 2,2′-azino-bis(3-ethylbenzothiazoline-6-sulfonic acid) enzymatic assay (ABTS%), and Lyco, 2,2-diphenyl-1-picrylhydrazyl-free radical scavenging assay (DPPH%). All analysis were conducted in triplicates.

#### 2.2.1 Photosynthetic pigments

Fresh leaf chlorophyll a and b and carotenoids were measured (Lichtenthaler and Buschmann, 2001). The fresh tissue was grounded in a mortar and pestles using 80% acetone. Photosynthetic pigment concentrations were measured in mg g^-1^ fresh weight (FW).

#### 2.2.2 Indole acetic acid

A known weight of fresh samples was obtained and extracted three times with 85% cold methanol (v/v) at 0°C to determine indole acetic acid concentration (IAA). At a wavelength of 530 nm, the developing color was spectrophotometrically quantified (Larsen et al., 1962).

#### 2.2.3 Free amino acids and proline

In g g^-1^ DW, free amino acids (AA) and proline (Pro) were extracted according to Vartanian et al. (1992). The ninhydrin reagent method was used to determine free amino acids (Yemm et al., 1955). Proline was measured using the method defined previously by Bates et al. (1973).

#### 2.2.4 Total soluble sugars

Total soluble sugars (TSS) in mg g^-1^ DW were extracted by immersing dry tissue in 10 ml 80% (v/v) ethanol overnight at 25°C with occasional shaking and centrifuging at 600 g. TSS was determined by boiling 0.1 mL of the ethanolic extract with 3.0 mL newly made anthrone (150 mg anthrone+100 mL 72% H_2_SO_4_) for ten minutes and scanning the cooled samples at 625 nm with a Spekol SpectrocololourimeterVEB Carl Zeiss (Yemm and Willis, 1956).

#### 2.2.5 Total carbohydrates

Total carbohydrates (TC) were determined (1971) by placing 0.2-0.5 g of dry plant tissue in a test tube and then adding 10 mL of sulphuric acid (1N). The tube was sealed and placed in a 100°C oven overnight. The solution was then filtered into a 100 mL measuring flask, and distilled water was added to the mark. Colorimetry was used to determine the total sugars (Dubois et al., 1956).

#### 2.2.6 Hydrogen peroxide

Leaf samples weighing 0.5 g were homogenized in 3 mL of 1% tri-chloroacetic acid (w/v) (TCA) to measure the concentration of hydrogen peroxide (H_2_O_2_) in nmol g^-1^ FW was determined (Velikova et al., 2000). A standard curve plotted in the range of 100 to 1000 μmol mL^-1^ was used to calculate hydrogen peroxide concentration.

#### 2.2.7 Lipid peroxidation

The levels of malondialdehyde (MDA) and total aldehydes were used to determine lipid peroxidation levels (ALD). Malondialdehyde and total aldehydes are products of lipid peroxidation, and their concentrations were determined using thiobarbituric acid reactive substrates (TBARS) (Hodges et al., 1999) with certain modifications.

#### 2.2.8 Assay of enzymes activities

Leaf tissues were homogenized in ice-cold phosphate buffer (50 mM, pH 7.8), then centrifuged for 15 minutes at 8,000 rpm and 4°C. The enzyme activities were determined right away using the supernatant.

A 0.5 mL of plant extract, 1 mL of 125 mM sodium carbonate, 0.4 mL of 25 μM NBT, and 0.2 mL of 0.1 mM EDTA were added to measure superoxide dismutase (SOD) activity (Chen and Wang, 2006). The peroxidase activity (POX, EC 1.11.1.7) was measured using Bergmeyer (1974).

The catalase activity (CAT, EC 1.11.1.6) was measured using spectrophotometry by following the reduction in absorbance at 240 nm. The 0.01 deduction in absorbance at 240 nm per minute was determined as one unit of CAT activity.

Fresh tissue was sliced into 2 mm slices and placed in ice-cold incubation media containing 3.0 mL of 0.05 M potassium phosphate buffer (pH: 7.8) and 3.0mL of 0.4M KNO_3_ solution to determine the activity of nitrate reductase (NR, EC 1. 7. 1. 1) (Jaworski, 1971). The activity of the NR was measured in nanomoles of nitrite per gram of plant fresh weight per hour (nM NO_2_/g FW/h).

#### 2.2.9 Measurement of non-enzymatic antioxidant

Ascorbic acid (AsA) was determined by grinding a fresh frozen leaf (0.2 g), extracting it, and homogenizing aliquots (10 g) in triplicate with a 50 mL solution mixture (3 percent w/v metaphosphoric acid + 8% acetic acid) (Helrich, 1990). On a dry weight basis, ascorbic acid concentration was given as µmol ascorbic 100 g^-1^.

Fully expanded and newly harvested wheat leaves (0.5 g) were homogenized with 2 mL of 2% metaphosphoric acid (v/v) solution and centrifuged for 10 minutes at 17,000 × g to determine glutathione content (GSH). Following Griffith (1980), standard curves were created utilizing samples and standards readings to quantify GSH concentrations on a dry weight basis in μ mol 100 g^-1^.

Total phenolic content (TPC) was extracted, and then 0.5 mL of the extraction was mixed with 0.5 mL Folin, shaken, and allowed to stand for 3 minutes. The optical density was measured using a UV-VIS spectrophotometer (Model DU 640B, Beckman, USA) at a wavelength of 725 nm (Diaz and Martin, 1972).

The aluminum chloride colorimetric method was used to quantify the total flavonoid content (TFC) of crude extract (Chang et al., 2002). A calibration curve was used to calculate the total flavonoid content, which was expressed as mg g^-1^ dry weight. Lycopene (Lyco) and β-carotene (B-Car) were measured as described (Nagata and Yamashita, 1992).

#### 2.2.10 Free radical scavenging activity

The free radical scavenging assay of 2,2-diphenyl-1-picrylhydrazyl (DPPH%) was measured by different plant extracts (Gyamfi et al., 1999), while 2,2′-azino-bis (3-ethylbenzothiazoline-6-sulfonic acid) enzymatic assay (ABTS%), ABTS cation radical was done (Nenadis et al., 2004).

### 2.3 Statistical analysis

Data were subjected to an analysis of variance for a factorial experiment in a completely randomized design (Gomez and Gomez, 1984), after testing for the homogeneity of error variances by Levene’s test (Levene, 1960) and after testing for normality distribution (Shapiro and Wilk, 1965). Tukey’s HSD (honestly significant difference) test was used to compare the differences between means at *P* ≤ 0.05. The statistical analysis was conducted using GenStat 19^th^ Edition (VSN International Ltd, Hemel Hempstead, UK). To assess the association between seed yield and each of the physiological and chemical characteristics, the correlation coefficient r was determined. Past software version 4.03 (2001) was used for performing Hierarchical cluster analysis.

## 3 Results

To achieve the objectives of this study, twenty-six traits were measured to examine the potential roles of aspartic acid (Asp) in increasing the tolerance in wheat plants grown under salt stress.

### 3.1 Changes in photosynthetic pigments and indoleacetic acid (IAA)

Results on the effect of Asp application on chlorophyll *a* (Chl *a*), chlorophyll *b* (Chl *b*), carotenoids (Car), total photosynthetic pigments (TPP), Chl *a*: Chl *b* ratio, as well as indoleacetic acid (IAA) in wheat plants grown under salinity stress (NaCl) for 60 days, are indicated in **Table 1**. Data showed that wheat plants were irrigated with salty water (30 and 60 mM) triggered a substantial reduction in the Chl *a*, Chl *b*, total pigments, and Chl *a*: Chl *b* ratio in addition to endogenous indole acetic acid in the leaves of wheat plants. Various salt concentrations resulted in a significant enhancement in carotenoid contents in comparison with plants irrigated with tap water (control). These reductions in response to salinity stress gradually decreased with increasing salinity levels from 30 to 60 mM. Saline water with 60 mM compared to control treatment caused a lessening in content of TPP and IAA by 26.7 and 19.7%, respectively.

**Table 1:** Effect of aspartic acid (Asp) on chlorophyll *a* (Chl *a*), chlorophyll *b* (Chl *b*), carotenoids (Car), total photosynthetic pigments (TPP), Chl *a*:Chl *b* ratio, and indoleacetic acid (IAA) in wheat plants grown under salt stress (NaCl) for 60 days.

Treatment		Chl *a*	Chl *b*	Car	TPP	Chl *a:* Chl *b*	IAA
		(mg g^-1^ FW)				ratio	(µg g^-1^ FW)
NaCl (mM)							
0		1.835^†^ a	0.633 a	0.317 c	2.785 a	2.89 a	6.88 a
30		1.350 b	0.511 b	0.395 b	2.256 b	2.64 b	6.37 b
60		1.123 c	0.404 c	0.514 a	2.041 c	2.80 a	5.53 c
Asp (mM)							
0.0		1.217 d	0.466 d	0.355 b	2.039 d	2.63 b	5.57 b
0.4		1.404 c	0.498 c	0.436 a	2.338 c	2.83 a	5.87 b
0.6		1.530 b	0.538 b	0.386 b	2.454 b	2.82 a	6.66 a
0.8		1.594 a	0.561 a	0.457 a	2.612 a	2.82 a	6.94 a
NaCl (mM)	Asp (mM)						
0	0.0	1.500 c	0.569 cd	0.270 f	2.339 cd	2.64 bc	6.19 cde
0	0.4	1.752 b	0.613 bc	0.325 ef	2.690 b	2.86 ab	6.50 bcd
0	0.6	2.014 a	0.660 ab	0.332 ef	3.005 a	3.05 a	7.33 a
0	0.8	2.075 a	0.690 a	0.343 ef	3.108 a	3.01 ab	7.51 a
30	0.0	1.173 ef	0.479 fg	0.359 def	2.012 e	2.45 c	5.54 efg
30	0.4	1.341 d	0.506 ef	0.487 bc	2.334 cd	2.65 bc	5.96 de
30	0.6	1.414 cd	0.520 def	0.324 ef	2.258 d	2.72 abc	6.83 abc
30	0.8	1.474 c	0.539 de	0.407 cde	2.420 c	2.74 abc	7.13 ab
60	0.0	0.979 g	0.351 h	0.435 bcd	1.765 f	2.79 abc	4.98 g
60	0.4	1.119 f	0.377 h	0.496 bc	1.991 e	2.97 ab	5.13 fg
60	0.6	1.162 ef	0.434 g	0.502 b	2.098 e	2.69 abc	5.80 def
60	0.8	1.233 e	0.453 g	0.622 a	2.308 cd	2.73 abc	6.19 cde

^†^Mean values within the same column for each trait with the same lower-case letter are not significantly different according to Tukey’s honestly significant difference (HSD) test at p ≤ 0.05. The results presented are from wheat plants harvested 75 days after sowing (DAS).

Foliar-applied treatments of wheat plants with various levels of Asp (0.4, 0.6, and 0.8 mM) significantly increased all mentioned traits, and the increase coincided with Asp increase level (**Table 1**). For example, Asp at 60 mM compared to control increased TPP and IAA by 28.1 and 24.6%, respectively. Interactive impacts were substantial in all studied parameters. It was revealed that foliar Asp applied at 60 mM under control salinity gave higher values for wholly studied parameters except for carotenoid. Application of Asp at 0.8 mM on plants irrigated with 60 mM NaCl resulted in improved traits compared to control. For example, TPP and IAA increased by 30.7 and 24.2%, respectively with plants (60 mM NaCl at 0.8 mM Asp) compared to control plants (60 mM NaCl at 0.0 mM Asp). This data indicated that wheat plants responded better to foliar Asp application under salinity than salinity at 60 mM NaCl (**Table 1**).

### 3.2 Changes in compatible solute accumulation and total carbohydrates

**Table 2** shows the effect of Asp application on compatible solute accumulation (amino acids; AA, proline; Pro, total soluble sugar; TSS, and total carbohydrates; TC) in wheat plants grown under salt stress (NaCl) for 60 days. Wheat plants irrigated with salty water in a concentration of 30 and 60 mM significantly increased AA, Pro, and TSS while decreasing TC compared to the control treatment. The increase reached about 14.2% compared to control, while the reduction in TC reached 6.4%. Foliar application with Asp caused a more significant enhancement in the studied parameters than the untreated control. The AA, Pro, TSS, and TC enhancement reached 20.2, 22.3, 20.2, and 5.4% in the application of Asp at 0.8 mM compared to control plants (0.0 mM Asp). At interaction effects and when the application of Asp at 0.8 mM on plants irrigated with 60 mM NaCl resulted in improved traits compared to control Asp under 60 mM NaCl. For example, AA and TC increased by 19.3 and 6.7%, respectively with plants (60 mM NaCl × 0.8 mM Asp) compared to control plants (60 mM NaCl × 0.0 mM Asp) (**Table 2**).

**Table 2:** Effect of aspartic acid (Asp) on compatible solute accumulation (amino acids; AA, proline; Pro, total soluble sugar; TSS, and total carbohydrates; TC) in wheat plants grown under salt stress (NaCl) for 60 days.

Treatment		AA	Pro	TSS	TC
		(µg g^-1^ DW)		(mg g^-1^ DW)	(g kg^-1^ DW)
NaCl (mM)					
0		245^†^ c	38.0 c	41.9 c	447 a
30		258 b	41.2 b	44.2 b	426 b
60		280 a	43.4 a	47.9 a	419 c
Asp (mM)					
0.0		235 d	36.5 c	40.3 d	419 d
0.4		256 c	40.0 b	44.1 c	428 c
0.6		270 b	42.4 ab	45.9 b	434 b
0.8		283 a	44.6 a	48.4 a	442 a
NaCl (mM)	Asp (mM)				
0	0.0	215 g	33.4 e	36.8 i	436 cd
0	0.4	243 f	37.6 cde	41.5 g	444 bc
0	0.6	254 e	39.4 b-e	43.5 f	452 ab
0	0.8	268 d	41.6 a-d	45.9 d	458 a
30	0.0	236 f	36.6 de	40.4 h	417 f
30	0.4	252 e	39.9 bcd	44.0 ef	425 ef
30	0.6	268 d	43.0 abc	44.8 e	425 ef
30	0.8	277 c	45.3 ab	47.4 c	437 cd
60	0.0	254 e	39.4 b-e	43.5 f	404 g
60	0.4	274 cd	42.4 a-d	46.8 cd	416 f
60	0.6	288 b	44.7 ab	49.3 b	423 ef
60	0.8	303 a	47.0 a	51.9 a	431 de

^†^Mean values within the same column for each trait with the same lower-case letter are not significantly different according to Tukey’s honestly significant difference (HSD) test at p ≤ 0.05. The results presented are from wheat plants harvested 75 days after sowing (DAS).

### 3.3 Changes in hydrogen peroxide, lipid peroxidation, and total aldehydes

The influence of Asp application on hydrogen peroxide (H_2_O_2_), lipid peroxidation (MDA), and aldehyde (ALD) under salt stress (NaCl) for 60 days is shown in **Table 3**. Wheat plants treated with salty water in 30 and 60 mM significantly increased H_2_O_2_, MDA, and ALD compared with those irrigated with distilled water, and the increase due to 60 mM NaCl compared to distilled water was about 7.6, 43.6, and 73.2% in H_2_O_2_, MDA, and ALD, respectively. Foliar application of Asp significantly reduced H_2_O_2_, MDA, and ALD compared with the untreated control, and this decrease was gradual with increasing the Asp concentration. A reduction in H_2_O_2_, MDA, and ALD was about 19.5, 24.4, and 43.8% due to the application of Asp at 0.8 mM compared to control plants (0.0 mM Asp). The interactive impact was found significant in H_2_O_2_, MDA, and ALD. Applying 0.8 mM Asp on plants irrigated with 60 mM NaCl reduced those traits compared to control Asp under 60 mM NaCl. Thus, H_2_O_2_, MDA, and ALD decreased by 16.9, 19.7 and 39.2%, respectively with plants (60 mM NaCl × 0.8 mM Asp) compared to control plants (60 mM NaCl × 0.0 mM Asp) (**Table 3**).

**Table 3:** Effect of aspartic acid (Asp) on hydrogen peroxide (H_2_O_2_), lipid peroxidation (MDA), and aldehyde (ALD) in wheat plants grown under salt stress (NaCl) for 60 days.

Treatment		H_2_O_2_	MDA	ALD
		(nmol g^-1^ FW)		
NaCl (mM)				
0		23.8^†^ c	45.8^†^ c	25.2 c
30		40.4 b	54.3 b	36.3 b
60		49.4 a	65.7 a	43.7 a
Asp (mM)				
0.0		44.2 a	64.1 a	44.9 a
0.4		37.8 b	57.2 ab	38.9 a
0.6		33.6 d	51.2 bc	31.4 b
0.8		35.6 c	48.5 c	25.2 b
NaCl (mM) Asp (mM)				
0	0.0	27.2 f	51.0 bc	28.2 cd
0	0.4	24.4 g	47.0 bc	28.5 cd
0	0.6	21.0 h	42.5 c	24.8 cd
0	0.8	23.0 gh	42.5 c	19.2 d
30	0.0	46.6 b	70.1 a	51.5 a
30	0.4	41.4 c	54.6 abc	39.5 abc
30	0.6	38.8 d	46.7 bc	31.2 cd
30	0.8	35.0 e	45.6 c	22.8 cd
60	0.0	59.2 a	71.4 a	54.8 a
60	0.4	47.8 b	69.9 a	48.5 ab
60	0.6	41.4 cd	64.5 ab	38.1 abc
60	0.8	49.2 b	57.3 abc	33.3 bcd

^†^Mean values within the same column for each trait with the same lower-case letter are not significantly different according to Tukey’s honestly significant difference (HSD) test at p ≤ 0.05. The results presented are from wheat plants harvested 75 days after sowing (DAS).

### 3.4 Changes in enzymatic antioxidants

The effect of exogenous Asp application on enzymatic antioxidants in wheat plants grown-up under various extents of salt stress (NaCl) for 60 days is shown in **Table 4**. Irrigated wheat plants with saline water with 30 and 60 mM significantly increased SOD, POX, CAT, and NR compared with those irrigated with distilled water, and the increase due to 60 mM NaCl compared to distilled water was about 40.5, 32.2, 29.4, and 37.8% in SOD, POX, CAT, and NR, respectively. Foliar application of Asp significantly enhanced the contents of SOD, POX, and CAT, while reducing NR compared with untreated control. An increase in SOD, POX, and CAT was about 19.7, 16.9, and 28.2% due to the application of Asp at 0.8 mM compared to control plants (0.0 mM Asp). However, 0.8 mM Asp significantly decreased NR by about 8.9% compared to control plants (0.0 mM Asp).

**Table 4:** Effect of aspartic acid (Asp) on enzymatic antioxidants (superoxide dismutase; SOD, peroxidase; POX, catalase; CAT, and nitrate reductase; NR) in wheat plants grown under salt stress (NaCl) for 60 days.

Treatment		SOD	POX	CAT	NR
			(U/min/g FW)		(nM NO_2_ g^-1^ FW)
NaCl (mM)					
0		27.5^†^ c	62.7 c	24.6 c	323 c
30		29.8 b	66.8 b	40.1b	342 b
60		38.6 a	82.8 a	56.5 a	445 a
Asp (mM)					
0.0		29.3 d	65.4 d	35.3 d	404 a
0.4		31.2 c	69.2 c	38.2 c	368 b
0.6		32.4 b	72.1 b	42.8 b	340 c
0.8		35.1 a	76.5 a	45.3 a	368 b
N NaCl (mM)	Asp (mM)				
0	0.0	25.8 h	56.5 g	24.8 hi	340 e
0	0.4	26.8 gh	61.9 f	23.7 i	321 f
0	0.6	27.9 fg	64.8 ef	24.3 hi	310 fg
0	0.8	29.5 ef	67.5 e	25.6 h	321 f
30	0.0	26.8 gh	63.6 f	32.2 g	381 d
30	0.4	28.9 ef	64.6 ef	35.7 f	348 e
30	0.6	30.6 e	67.5 e	42.2 e	302 g
30	0.8	33.1 d	71.6 d	50.2 d	336 e
60	0.0	35.3 c	76.1 c	48.9 d	491 a
60	0.4	37.8 b	81.0 b	55.2 c	435 b
60	0.6	38.8 b	84.0 b	61.7 a	407 c
60	0.8	42.7 a	90.2 a	60.0 b	447 b

^†^Mean values within the same column for each trait with the same lower-case letter are not significantly different according to Tukey’s honestly significant difference (HSD) test at p ≤ 0.05. The results presented are from wheat plants harvested 75 days after sowing (DAS).

The interaction effects were significant in SOD, POX, CAT, and NR. Application of 0.8 mM Asp on plants irrigated with 60 mM NaCl increased those traits compared to control Asp under 60 mM NaCl. Thus, SOD, POX, and CAT increased by 20.8, 18.5, and 22.7%, respectively, with plants grown under 60 mM NaCl and treated with0.8 mM Asp, compared to control plants (60 mM NaCl × 0.0 mM Asp) (**Table 3**). However, the application of 0.8 mM Asp significantly decreased NR by about 9.1% compared to control plants (0.0 mM Asp) (**Table 4**).

### 3.5 Changes in non-enzymatic antioxidants

Results related to the influence of aspartic acid (Asp), on non-enzymatic antioxidants (ascorbate; AsA, glutathione (GSH), total phenolic content (TPC), total flavonoid content; TFC, beta carotene; B-Car, lycopene; Lyco) under salt stress (NaCl) for 60 days are presented in **Table 5**. Saline water applied in a concentration of 30 and 60 mM significantly increased AsA, GSH, and TPC, while decreasing TFC, B-Car, and Lyco compared with those irrigated with distilled water. The increase in AsA, GSH, and TPC reached about 28.6, 18.7, and 61.5% compared to control, while the reduction in AsA, GSH, and TPC reached 19.7, 18.3, and 23.9%, respectively. Foliar application with various levels of aspartic acid (0.4, 0.6, and 0.8 mM) significantly increased AsA, GSH, TPC, TFC, B-Car, and Lyco compared with those irrigated with distilled water. The increase in AsA, GSH, TPC, TFC, B-Car, and Lyco reached about 20.4, 10.6, 38.1, 24.6, 7.4, and 24.5%, respectively, and the increase coincided with the Asp increase level. The interaction effects were significant in AsA, GSH, TPC, TFC, B-Car, and Lyco. Application of 0.8 mM Asp on plants irrigated with 60 mM NaCl increased those traits compared to control Asp under 60 mM NaCl. Thus, AsA, GSH, and TPC, TFC, B-Car and Lyco increased by 1.3, 10.6, 37.9, 24.2, 5.0, and 14.0%, respectively with plants (60 mM NaCl × 0.8 mM Asp) compared to control plants (60 mM NaCl × 0.0 mM Asp) (**Table 5**).

**Table 5:** Effect of aspartic acid (Asp) on non-enzymatic antioxidants (ascorbate; AsA, glutathione (GSH), total phenolic content (TPC), total flavonoid content; TFC, beta carotene; B-Car, lycopene; Lyco) in wheat plants grown under salt stress (NaCl) for 60 days.

Treatment		AsA	GSH	TPC	TFC	B-Car	Lyco
		(µmol ascorbic100 g^-1^ DW)	(µmol glutathione100 g^-1^ DW)	(mg 100 g^-1^ DW)			
NaCl (mM)							
0		258^†^ c	139 c	40.9 c	17.3 a	0.474 a	0.423 a
30		307 b	149 b	54.8 b	16.0 b	0.444 b	0.370 b
60		331 a	165 a	66.1 a	13.9 c	0.387 c	0.322 c
Asp (mM)							
0.0		276 d	141 d	44.6 c	14.0 b	0.400 d	0.327 d
0.4		289 c	150 c	53.2 b	14.8 b	0.447 b	0.364 c
0.6		297 b	157 a	56.3 ab	16.8 a	0.465 a	0.389 b
0.8		332 a	156 b	61.6 a	17.5 a	0.429 c	0.407 a
NaCl (mM)	Asp (mM)						
0	0.0	215 j	131 g	37.3 f	15.6 cde	0.431 e	0.360 e
0	0.4	236 i	139 f	41.2 ef	16.4 bcd	0.490 b	0.403 c
0	0.6	241 h	144 e	43.1 def	18.5 a	0.507 a	0.427 b
0	0.8	339 a	143 e	42.1 ef	18.9 a	0.468 c	0.503 a
30	0.0	288 g	137 f	41.8 ef	14.0 efg	0.407 fg	0.331 fg
30	0.4	297 f	150 d	51.9 c-f	15.0 de	0.453 d	0.365 e
30	0.6	313 e	155 c	58.0 bcd	17.2 abc	0.477 c	0.397 cd
30	0.8	328 c	153 cd	67.3 abc	18.0 ab	0.440 e	0.388 d
60	0.0	325 d	155 c	54.7 b-e	12.6 g	0.361 i	0.290 h
60	0.4	334 b	162 b	66.4 abc	12.9 fg	0.398 g	0.325 g
60	0.6	338 a	173 a	67.8 ab	14.6 def	0.410 f	0.343 f
60	0.8	329 c	171 a	75.4 a	15.6 cde	0.379 h	0.330 fg

^†^Mean values within the same column for each trait with the same lower-case letter are not significantly different according to Tukey’s honestly significant difference (HSD) test at p ≤ 0.05. The results presented are from wheat plants harvested 75 days after sowing (DAS).

### 3.6 Changes in free radical scavenging activity (DPPH%) and enzymatic scavenging activity (ABTS%)

**Table 6**, shows the effect of aspartic acid (Asp) on DPPH%, 2,2′-azino-bis, and ABTS% in wheat plants grown under salt stress (NaCl) for 60 days. Irrigation of wheat plants with saline water concentrations of 30 and 60 mM significantly increased ABTS%, while decreased DPPH%. The increase in ABTS% reached about 29.0% compared to control, while the reduction in DPPH% reached 23.3%, respectively. Foliar application with Asp caused a more significant enhancement in studied parameters than the untreated control. The increase in DPPH% and ABTS% reached 43.1 and 16.3% in the application of Asp at 0.8 mM compared to control plants (0.0 mM Asp). The interactive impact was significant in DPPH% as well as ABTS%. Applying 0.8 mM Asp on plants irrigated with 60 mM NaCl increased those traits compared to control Asp under 60 mM NaCl. Thus, DPPH% and ABTS% increased by 7.7 and 6.0%, respectively with plants (60 mM NaCl × 0.8 mM Asp) compared to control plants (60 mM NaCl × 0.0 mM Asp) (**Table 6**).

**Table 6:** Effect of aspartic acid (Asp) on 2,2-diphenyl-1-picrylhydrazyl-free radical scavenging assay (DPPH%) and 2,2′-azino-bis (3-ethylbenzothiazoline-6-sulfonic acid) enzymatic assay (ABTS%) in wheat plants grown under salt stress (NaCl) for 60 days.

Treatment		DPPH	ABTS
		**(%)**	
NaCl (mM)			
0		59.3^†^ a	35.3 c
30		53.5 b	40.4 b
60		45.5 c	45.5 a
Asp (mM)			
0.0		49.6 d	37.1 d
0.4		52.9 c	39.3 c
0.6		53.5 b	42.0 b
0.8		55.1 a	43.1 a
NaCl (mM)	Asp (mM)		
0	0.0	54.4 cd	33.1 e
0	0.4	58.8 b	34.3 e
0	0.6	61.6 a	37.1 d
0	0.8	62.5 a	36.6 d
30	0.0	50.5 e	34.7 e
30	0.4	54.6 c	38.2 d
30	0.6	53.4 d	41.7 c
30	0.8	55.3 c	46.9 ab
60	0.0	44.0 h	43.3 c
60	0.4	45.2 gh	45.3 b
60	0.6	45.4 g	47.4 a
60	0.8	47.4 f	45.9 ab

^†^Mean values within the same column for each trait with the same lower-case letter are not significantly different according to Tukey’s honestly significant difference (HSD) test at p ≤ 0.05. Measurements were done after 75 days after sowing.

### 3.7 Changes in growth and biomass yield of the wheat plant

Effect of aspartic acid (Asp) on shoot length (SL), total leaves area per plant (LA), and shoot dry weight per plant (SDW) in wheat plants grown-up under salt stress (NaCl) for 60 days **Table 7**. Wheat plants irrigated with salty water at 30 and 60 mM levels significantly decreased SL, LA, and SDW. For example, saline water at 60 mM NaCl decreased SDW by 2.6 folds compared to control distilled water. Foliar application with Asp caused a more significant enhancement in the studied parameters compared to untreated control. The increase in SDW reached 66.2% in the application of Asp at 0.8 mM compared to control plants (0.0 mM Asp). The interactive impacts were found significant in SL, LA, and SDW. Applying 0.8 mM Asp on plants irrigated with 60 mM NaCl increased those traits compared to control Asp under 60 mM NaCl. Thus, SDW increased by 2-folds with plants (60 mM NaCl × 0.8 mM Asp) compared to control plants (60 mM NaCl × 0.0 mM Asp) (**Table 7**).

**Table 7:** Effect of aspartic acid (Asp) on shoot length (SL), total leaves area per plant (LA), and shoot dry weight per plant (SDW) in wheat plants grown under salt stress (NaCl) for 60 days.

Treatment		SL	LA	SDW
		(cm)	(dm^2^)	(g plant^-1^)
NaCl (mM)				
0		56.7^†^ a	1.79 a	2.24 a
30		53.4 b	1.30 b	1.63 b
60		36.6 c	0.69 c	0.86 c
Asp (mM)				
0.0		44.0 c	0.93 d	1.17 d
0.4		48.8 b	1.19 c	1.49 c
0.6		52.1 a	1.36 b	1.71 b
0.8		50.8 a	1.55 a	1.94 a
NaCl (mM)	Asp (mM)			
0	0.0	52.5 cd	1.23 ef	1.54 ef
0	0.4	56.9 ab	1.77 c	2.21 c
0	0.6	58.1ab	1.94 b	2.43 b
0	0.8	59.4 a	2.23 a	2.79 a
30	0.0	48.8 d	1.13 f	1.41 f
30	0.4	55.6 abc	1.25 ef	1.56 ef
30	0.6	55.0 bc	1.31 e	1.64 e
30	0.8	54.4 bc	1.51 d	1.89 d
60	0.0	30.6 g	0.44 h	0.55 h
60	0.4	33.8 g	0.56 h	0.70 h
60	0.6	43.1 e	0.84 g	1.05 g
60	0.8	38.8 f	0.91 g	1.13 g

^†^Mean values within the same column for each trait with the same lower-case letter are not significantly different according to Tukey’s honestly significant difference (HSD) test at p ≤ 0.05. Measurements were done after 75 days after sowing.

### 3.8 Correlation matrix

Pearson’s correlation coefficients among shoot dry weight and entirely studied parameters of wheat plant foliar applied with four levels of aspartic acid grown under three groups of 0, 30, or 60 mM NaCl salt stress for 60 days (**Table 8**). The consequences of the study revealed that there was found a significantly correlated relationship between SDW and each of Chl *a*, Chl *b*, TPP, IAA, TC, TFC, B-Car, Lyco, DPPH, SL, and LA, while significant negative associations were reported between SDW and each of Car, POX, SOD, CAT, NR, H_2_O_2_, MDA, and ALD.

**Table 8:** Pearson’s correlation coefficients among shoot dry weight and all measured traits of whaet plants foliar sprayed with 4 levels of aspartic acid grown under three levels of 0, 30, or 60 mM NaCl salt stress for 60 days (n = 12).

**Traits**	**Chl a**	**Chl b**	**Car**	**TPP**	**Chl a/b**	**IAA**	**AA**
SDW	0.960	0.981	-0.579	0.946	0.383	0.906	-0.195
p-value	0.00	0.00	0.05	0.00	0.22	0.00	0.54
**Traits**	**Pro**	**TSS**	**TC**	**POX**	**SOD**	**CAT**	**NR**
SDW	-0.156	-0.199	0.926	-0.572	-0.616	-0.729	-0.854
p-value	0.63	0.54	0.00	0.05	0.03	0.01	0.00
**Traits**	**AsA**	**GSH**	**TPC**	**TFC**	**B-Car**	**Lyco**	**H2O2**
SDW	-0.355	-0.545	-0.550	0.906	0.861	0.945	-0.897
p-value	0.26	0.07	0.06	0.00	0.00	0.00	0.00
**Traits**	**MDA**	**ALD**	**DPPH**	**ABTS**	**SL**	**LA**	**SDW**
SDW	-0.879	-0.831	0.977	-0.544	0.910	1.000	1.000
p-value	0.00	0.00	0.00	0.07	0.000	0.00	

p-value, probability value (a p-value ≤ 0.05 is statistically significant); Chl a, chlorophyll a, Chl b, chlorophyll b; Car, carotenoids; TPP, total photosynthetic pigments; Chl a/b, Chl a:Chl b ratio; IAA, indoleacetic acid; AA, amino acids, Pro, proline; TSS, total soluble sugar; TC, total carbohydrates; SOD, superoxide dismutase; POX, peroxidase; CAT, catalase; NR nitrate reductase; AsA, ascorbate; GSH, glutathione; TPC, total phenolic content; TFC, total flavonoid content; B-Car, beta carotene; Lyco, lycopene; H_2_O_2_, hydrogen peroxide; MDA, lipid peroxidation; ALD, aldehyde; DPPH, 2,2-diphenyl-1-picrylhydrazyl-free radical scavenging assay; ABTS 2,2′-azino-bis enzymatic assay; SL, shoot length; LA, total leaves area per plant; SDW, shoot dry weight per plant.

### 3.9 Cluster analysis

**Figure 1:**
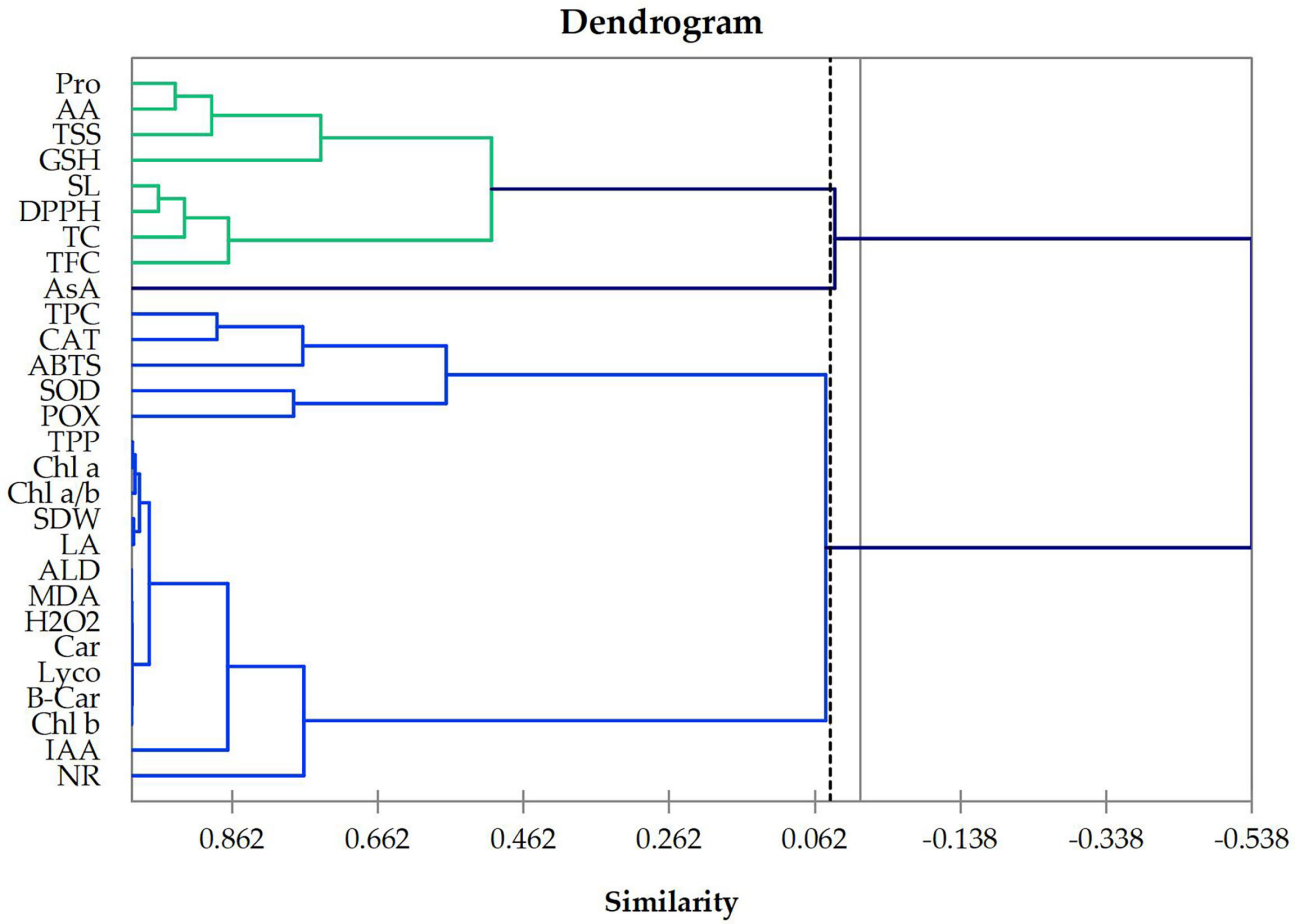
Dendrogram showing the distance among 28 wheat traits based on 12 treatments on wheat plants foliar sprayed with 4 levels of aspartic acid grown under salt stress (NaCl) for 60 days. Chl *a*, chlorophyll *a*; Chl *b*, chlorophyll *b*; Car, carotenoids; TPP, total photosynthetic pigments; Chl a/b, Chl a: Chl b ratio; IAA, indoleacetic acid; AA, amino acids, Pro, proline; TSS, total soluble sugar; TC, total carbohydrates; SOD, superoxide dismutase; POX, peroxidase; CAT, catalase; NR nitrate reductase; AsA, ascorbate; GSH, glutathione; TPC, total phenolic content; TFC, total flavonoid content; B-Car, beta carotene; Lyco, lycopene; H_2_O_2_, hydrogen peroxide; MDA, lipid peroxidation; ALD, aldehyde; DPPH, 2,2-diphenyl-1-picrylhydrazyl-free radical scavenging assay; ABTS 2,2′-azino-bis(3-ethylbenzothiazoline-6-sulfonic acid) enzymatic assay; SL, shoot length; LA, total leaves area per plant; SDW, shoot dry weight per plant.

Cluster analysis is an effective tool for classifying objects into groups. The cluster analysis was used as an efficient procedure to emerge the structural relationships among tested traits and provides a hierarchical classification among them. In the present work, based on Euclidean distance, the tested traits were classified according to 12 treatments on wheat plants sprayed with four levels of Asp grown under three groups of 0, 30, or 60 mM NaCl salt stress for 60 days and were discriminated as shown in dendrogram graph (**Figure 1**). The dendrogram for all studied traits, printed and plotted (with better resolution) in **Figure 1**, shows that the AA, Pro, TSS, TC, GSH, TFC, DPPH, and SL are most closely related in one class, while Chl *a*, Chl *b*, Car, TPP, Chl *a*/*b*, IAA, POX, SOD, CAT, NR, TPC, B-Car, Lyco, H_2_O_2_, MDA, ALD, ABTS, LA, and SDW are most correlated traits in the second class. However, the third class included only the trait that is AsA.

### 3.10 Response curve of shoot dry weight (SDW) to different application levels of aspartic acid (Asp)

**Figure 2:**
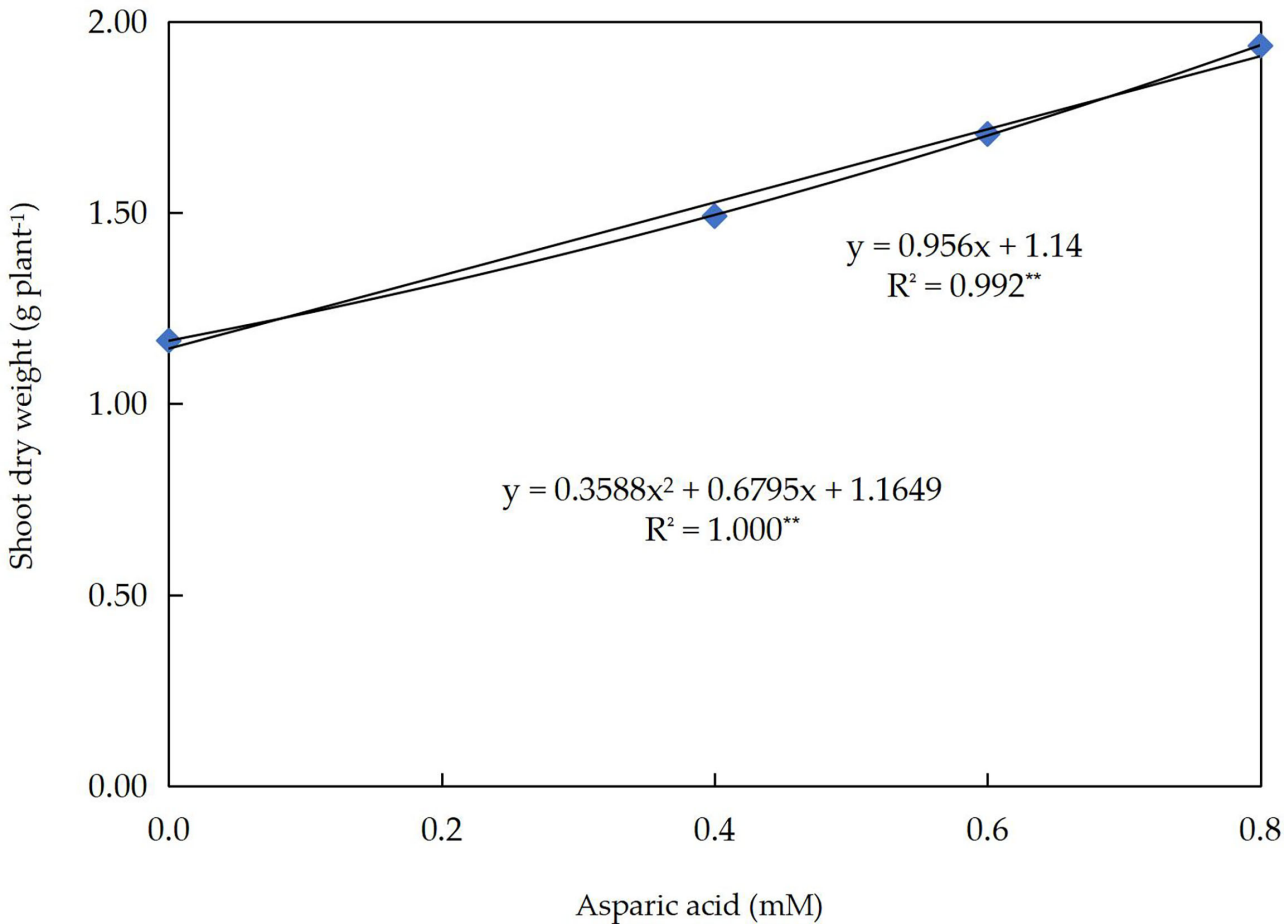
The response curve of shoot dry weight (g plant^–1^) to different application levels of aspartic acid (mM) in wheat plants grown under three levels of 0, 30, or 60 mM NaCl salt stress for 60 days.

Linear and quadratic responses of wheat shoot dry weight (g plant^–1^) to different application levels of Asp in wheat plants grown under three groups of 0, 30, or 60 mM NaCl for 60 days are shown in **Figure 2**. The R^2^ value has enhanced from 99.2% (linear) to 100% (quadratic). This indicates that the quadratic regression model explicates 100% of the variation in shoot dry weight yields. With an increased Asp application level of by1.0 mM, the SDW was probable to upsurge by 956 mg per plant. In the quadratic curve, if Asp is foliar sprayed at a level of 0.95 mM, the SDW is expected to be 2.13 g plant^-1^.

### 3.11 Treatment x Traits (TT) biplot

**Figure 3:**
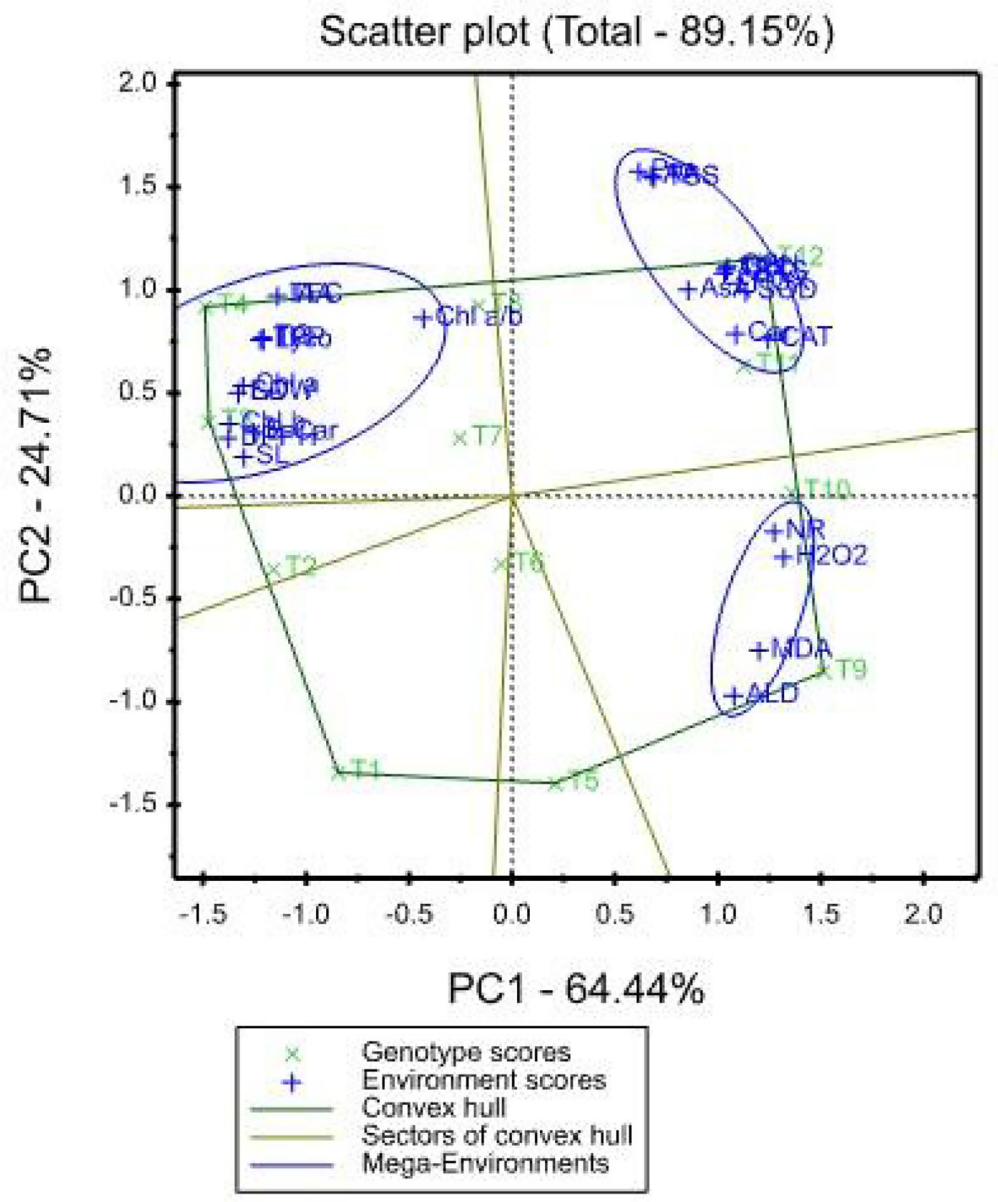
Polygon-view of TT biplot showing which factorial treatments of saline irrigation and aspartic acid had the highest values for which traits.

**Figure 4:**
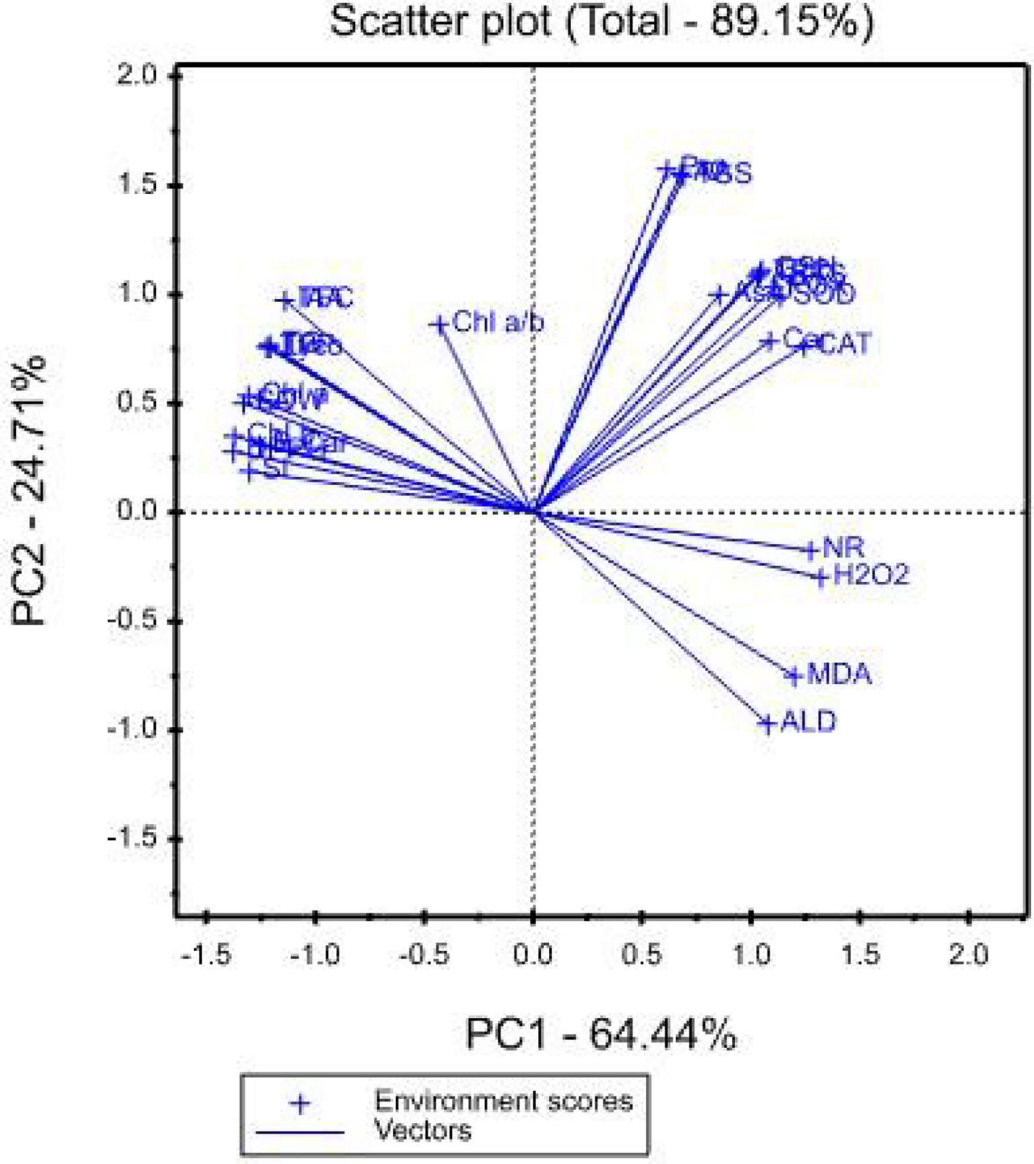
Vector view of TT biplot showing the interrelationship among measured traits of the wheat plant.

The mean values of the effects of saline irrigation treatment and four foliar applications of aspartic acid on the studied traits were graphically summarized in a polygon view, as shown in **Figure 3**. In addition, **Figure 4**, shows the vector view of the TT biplot showing the interrelationship among measured traits of the wheat plant. The polygon view of the TT biplot distinguishes the factorial treatments of saline irrigation and foliar sprayed aspartic acid, giving the highest values for one or more traits. In these results, the first three principal components have eigenvalues greater than 1. The first principal component accounts for 64.44% of the total variance. The variables that correlate the most with the first principal component (PC1) are Chl *b* (0.229), DPPH (0.229), SDW (0.222), LA (0.222), Chl *a* (0.218), SL (0.217), B-Car (0.209), TPP (0.204), Lyco (0.201), TC (0.201), TFC (0.190), and IAA (0.190). The first principal component is negatively correlated with all other variables. The first four principal components explain 95.37% of the variation in the data, while the first three explain 93.18% of the variation. The first and second principal components (PC) explained 64.44 and 24.71%, respectively. Therefore, the principal components (PC) analysis based on the TT biplot method (**Figure 3**) explained about 89.15% of the observed variation for the measured traits of wheat across saline irrigation and aspartic acid foliar application treatments. These results indicated that the two biplot graphs were characterized by a goodness of fit model. The two biplot graphs can explain sufficient amounts of the total variation of more than 89.15% of the treatment x trait pattern data. Polygon graph of aspartic acid and saline irrigation treatment combination - won- where-for wheat traits. In addition, T12 was the vertex treatment for specific studied traits followed by T11, T10, and T9.

## 4 Discussion

Amino acids present in the plants are engaged in primary and secondary metabolism and other cellular enzymatic activities as constituents of enzymes such as aminotransferases, dehydrogenases, lyases, and decarboxylases. As a result, they can affect a variety of phenological and physiological processes, including plant vegetative development, seed germination, fruit maturation, signaling and activation of defense systems against abiotic and biotic stresses by performing osmotic adjustment and deactivation of reactive oxygen species. (Teixeira et al., 2017). However, the application of bio-stimulant has become more effective in improving crop agronomic performance and they have to be explicitly formulated for crop varieties. It is necessary to understand the function of each amino acid in each crop, to identify antagonistic, synergistic, or neutral effects among amino acid mixtures, and to determine doses and application times. Our study found that foliar application of amino acids (Asp) can affect plant vegetative development and improve plant growth under salt stress. Moreover, aspartic acid enhanced the heat tolerance of perennial ryegrass (Lei et al., 2022).

Chloroplasts are the main target of reactive oxygen species under salinity stress, which cause the degradation of thylakoid membranes and photosynthetic pigments (Taiz and Zeiger, 2010). Salinity stress reduced photosynthetic pigments compared to unstressed plants (**Table 1**). These study outcomes are like those found in wheat (Saddiq et al., 2021), soybean (Sadak et al., 2020), and common bean (Rady et al., 2019). The inhibitory effect might be due to the closing of stomata and inhibiting CO_2_ fixation process as well as decreasing the transport of electrons *via* photosynthesis. In general, the decrease of photosynthetic products in plants grown under salinity stress is due to less development or faster degradation of photosynthetic pigments in the leaf cells (Ashraf, 2003). In addition, it was stated that subjecting the plant to salinity stress caused enhanced proline biosynthesis that led to the less effective usage of glutamate as a precursor in biosynthesizing chlorophyll molecules (Nazarbeygi et al., 2011). The high carotenoid contents under salinity stress can be explained by carotenoids’ role as free radical scavengers in leaf cells. Therefore, carotenoids in the wheat plant may cause enhancement in their capacity to reduce damages caused due to reactive oxygen species, which in turn enhances chlorophyll a and b contents in wheat plants.

This work reported the induced effect of Asp treatment on the wheat plant under control and salinity-stressed conditions. In this regard, it was reported that Asp and cysteine treatments increased photosynthetic pigments concentration in soybean (Sadak et al., 2020). Moreover, amino acids have a chelating influence on macronutrients such as Mg, that regulate the uptake and the transfer of all types of nutrients within the plant easier as a result of its impacts on the permeability of cells membranes (Marschner, 1995).

Salinity stress caused a significant decrease in the IAA concentration in new leaves of wheat compared with the control unstressed plants (**Table 1**). This decrease may be due to enhanced destruction of IAA by IAA oxidase (Bano and Yasmeen, 2010). Foliar application of Asp, in most cases, significantly enhanced IAA contents under un-stressed wheat plants and salinity-stressed plants with improving Asp levels as compared with corresponding controls. These increases in IAA correspond with enhancement in the growth rate and net assimilation rate (**Table 7**). They may cause attribution to the initiation in the cell divisions and/or cell expansion (Orabi and Sadak, 2015).

Different environmental stresses, such as salinity, cause an imbalance in plant hormone levels, leading to the accumulation of growth inhibitors and decreased growth promoters, such as IAA. This imbalance is a factor that activates stress-associated genes, responding with the accretion of several compatible solutes, for example, free amino acids, proline, as well as total soluble sugar content. Wheat plants irrigated with salty water produced significant free amino acids, proline, and total soluble sugar concentrations. The obtained results were confirmed for the other crops, like flax (El-Bassiouny and Sadak, 2015). The enhancements in the concentration of two main organically based osmolytes (proline as well as total soluble sugar) could help the plants to control the osmotic potential of cells, which resulted in the improvement of water absorption, as well as nutrients, transfer under the salinity stress condition (Oraki and Aghaalikhana, 2012). The proline is complicated in protecting the cellular structure and enzyme structure by oxidation damage and acts as scavengers for the free radicals (Rady et al., 2015). In the present research, foliar treatment with Asp caused a gradual increase in the studied compatible solutes. It confirmed the positive role of amino acids on the above-mentioned compatible solutes using methionine in soybean cultivation (Bakhoum et al., 2019). This positive role might be since Asp is a nitrogen source and is used in the biosynthetic process of other amino acids. All amino acids are considered growth-promoting factors and act as the main structures of all enzyme proteins.

Furthermore, enzymes are substantially involved in major metabolism procedures (El-Awadi et al., 2019). In the present research, salinity stress-triggered to a significant reduction in the total carbohydrate concentration of wheat plants. Similar findings were obtained for different crops. It was reported that total carbohydrate concentration was reduced in soybean and faba bean plants (Rady et al., 2015; Sadak and Abdelhamid, 2015), due to the reduction in photosynthetic pigments, also observed in the present study (**Table 1**). Carbohydrate changes in plant leaves are significant for physiological processes such as photosynthesis, translocation, and respiration (Asao and Ryan, 2015). Stress due to salty water reduced chlorophyll a and b concentrations in the leaves, causing a lessening of the photosynthesis process. Subsequently, lessened carbohydrate accumulations in matured leaves could consequently lessen carbohydrates transportation toward the developing seeds (Elewa et al., 2017). Conversely, the stimulation impact of Asp treatments on the carbohydrate concentrations may result from the enhancement in leaf photosynthesis pigments (**Table 1**).

Salinity stress increases the accumulation of ROS in cell constituents, which can negatively affect the chemical structure as well as the functioning of proteins, nucleic acids, lipids, and other crucial compounds (Apel and Hirt, 2004; Gill and Tuteja, 2010). Our results of H_2_O_2_, MDA, and aldehydes contents show that stress due to salts instigated significant enhancement of the contents of mentioned compounds (**Table 3**). Others support these results wherein salinity stress increased H_2_O_2_ and MDA levels in the sunflower plant (Abd El-Hameid and Sadak, 2020).

Lipid peroxidation is generally considered a measuring parameter of saltiness persuaded oxidation, based on stress and plant sensitivity due to salts’ harmful effects. In addition, the ultimate product of membrane lipid peroxidation is malondialdehyde (MDA). When MDA attaches to a protein, it causes cross-linking between the molecules. The cross-linking polymerization of structural and enzyme proteins, in particular, is particularly damaging to the plasma membrane. As a result, MDA levels may reflect the extent of tissue cell membrane damage (Li et al., 2009). The MDA content was reduced after treatment with exogenous aspartic acid in this study, showing that spraying aspartic acid had some protective effects on the cell membrane of wheat leaves under salt stress. Aldehydes and MDA are usually the last formed products of lipid peroxidation. Plant survival under stress requires two critical mechanisms: increasing antioxidant enzyme activity and accumulating osmotic regulators. Plants create reactive oxygen radicals with a high oxidative capacity that can disrupt the structure and function of many functional molecules in cells when stressed. As a result, scavenging reactive oxygen species is critical for maintaining the cell membrane’s health (Tian et al., 2005). According to previous research, SOD, POD, and CAT are powerful protective enzymes for ROS scavenging. They play a crucial role in avoiding membrane lipid peroxidation, delaying senescence, and maintaining normal growth and development in plants. SOD, POD, and CAT activity will rise in plants under mild stress-induced conditions, enhancing their stress resistance (Wang et al., 2011). In this study, the activity of SOD, POD, and CAT of leaves was decreased significantly under salt stress. After treatment with exogenous aspartic acid, SOD, POX, and CAT of wheat plants were increased considerably.

In our study, salinity stress induced increased levels of ROS. Nevertheless, plants can deal with such stress by enhancing the formation of the antioxidant metabolites, comprising enzymes such as SOD, POX, CAT, and NR (Schutzendubel and Polle, 2002). Our results showed that salinity stress increased the above-mentioned enzyme activity and these results are supported by those obtained on faba bean (El-Awadi et al., 2017). Moreover, exogenous application of Asp significantly enhanced the concentrations of SOD, POX, CAT, and NR in the wheat plant (**Table 4**).

The enhancement in peroxidase concentration, which was utilized as a biomarker of stress conditions in plants, could be a result of the raised concentration of POX localized in the cell walls, as stated in rice plants under stressful circumstances. Meanwhile, it contributes to the phenol as well as lignin formation process, instigating a corporeal blockade in toxic causing pollutants (Kose et al., 2011). Concerning SOD and NR enzymes (Ardebili et al., 2012). The activities of antioxidative enzymes play a critical role in ROS scavenging, and consequently, their stimulus might include enhancement of the salinity stress resistance and postponement of senescence in plants (Alscher et al., 2002). An upsurge in the activities of antioxidative enzymes by saltness stresses accumulating a protective mechanism to reduce the oxidative damage of cell components (Kose et al., 2011). More activities of these enzymes were recorded in plants treated with Asp and irrigated with different levels of saline water. It was reported that more concentrations of antioxidative enzymes such as CAT, POX, and PPO resulted in lowering of H_2_O_2_ formation, peroxidation of lipids, as well as more membrane solidity (Alscher et al., 2002). These enzymes are intricate in the exclusion of H_2_O_2_ from stressed cells. In this regard, it was confirmed that exogenous application of Asp significantly increased the specific activity of antioxidant enzymes of tomato plants in stressed plants. This might reduce the injurious effect of salt and which reacts with H_2_O_2_ and maintain the membrane integrity (Akladious and Abbas, 2013). Moreover, significant increases in contents of SOD, CAT, and POX as a result of salinity stress or proline, or when collected together, were reported (Sadak and Abdelhamid, 2015).

Salt stress damage in plants is characterized by the loss of chlorophyll and the generation of reactive oxygen species (ROS), resulting in lipid peroxidation (Sadak and Abdelhamid, 2015). In this study, exogenous application of Asp allowed plants to maintain lower ROS (H_2_O_2_) production and MDA content while higher chlorophyll content and antioxidant enzyme (SOD, POX, and CAT) activities. The favorable effects of Asp on the activity of antioxidant enzymes (SOD, POD, CAT, and APX) in rice (*Oryza sativa*) have been linked to Asp-mediated inhibition of cadmium-induced oxidative stress (Rizwan et al., 2017a). According to the findings, Asp significantly reduced salt damage and improved salt tolerance in wheat by reducing chlorophyll loss and activating enzymatic antioxidants to minimize oxidative damage caused by salt stress.

Concerning the non-enzymatic antioxidants in wheat plants under different saline water levels, the current results revealed a significant increase in ascorbic acid, glutathione, and total phenolics contents, while total flavonoids, B-carotene, and lycopene contents decreased compared with control (**Table 5**). The lycopene acts as a precursor of β-carotene, a fat-soluble carotenoid and exhibits two-fold higher antioxidation activity than β-carotene (Sandmann, 1994). Long-chain conjugated double bonds (polyene chains) with an ability to quench free radicals and present in lycopene are mainly attributed to its potential antioxidant ability (Britton, 1996). Another important function performed *via* lycopene is cell signaling and communication (Zhang et al., 1991), hormonal and immune response modulation, and role in metabolic pathways (Astorg et al., 1997). The present research measured the non-enzymatic antioxidant potential of the wheat plant, moderated by lycopene, β-carotene, flavonoids, and total antioxidant activity, which changed in response to Asp foliar treatment and salinity stress. Lycopene, as well as β-carotene, are usually considered natural antioxidative agents which act as utmost effective singlet oxygen quenchers *in vitro* among common carotenoids (Di Mascio et al., 1989). Some studies showed that β-carotene was substantially reduced under salt stress conditions in tomato fruit (Dorais, 2000). Moreover, under water stress, an adverse influence on lycopene and β-carotene accretion in the period of tomato ripening was reported (Ali and Ismail, 2014). Carotenoids are closely connected with the photosynthetic process as a part of the sunlight-capturing system. Moreover, it is well recognized that salinity stress overwhelms to photosynthetic process (Chartzoulakis and Klapaki, 2000). Therefore, under the dominant investigational circumstances, the reduction in lycopene and β-carotene concentrations was related to the decrease in the photosynthetic process under salinity. A possible explanation would be that salinity may inhibit or up-regulate the biosynthetic pathway of carotenoids *via* inhibition of the genes encoding enzymes related to lycopene and β-carotene formation (Dumas et al., 2003).

Moreover, salt stress caused an inhibition in the expression of the gene-encoded lycopene β-cyclase, the enzyme that converts lycopene to β-carotene (Babu et al., 2011). Aspartic acid treatments caused a significant enhancement in lycopene concentration, which was linked with a substantial enhancement in β-carotene (**Table 5**). Flavonoids, including flavones, flavanols, and condensed tannins, are plants’ secondary metabolites, the antioxidant activity of which depends on the presence of free OH groups, especially 3-OH. Plant flavonoids have antioxidant activity *in vitro* and also act as antioxidants *in vivo* (Geetha et al., 2003). Spraying wheat plants with Asp resulted in a significant increase in flavonoids contents compared to untreated plants. These observations revealed that the Asp may be an inducer for the biosynthesis of secondary metabolites (flavonoids), which acted as oxygen scavengers to reduce oxidative stress and increase wheat plant growth (**Table 5**).

Salinity stress caused significant decreases in the antioxidant activity of DPPH radical scavenging capacity of wheat plants (**Table 6**). Foliar treatment of wheat plants with different concentrations of Asp caused increases in the antioxidant activity of DPPH% and ABTS% compared with control plants (**Table 6**). It was suggested that high antioxidant activity and phenolic components have been reported in wheat and wheat-based food products, indicating that wheat may serve as an excellent dietary source of natural antioxidants in the human diet, causing better disease prevention and health promotion (Yu et al., 2002).

Higher plants influence the formation of secondary metabolites, for example, phenols that have an antioxidant agent for hunting reactive oxygen species. All phenolic composites protect cells and guard cells from potential oxidation impairment; upsurge the solidity of cell membranes in the plant cell (Burguieres et al., 2007). This enhancement might result from the entire phenols’ role to significantly impacting the plant’s metabolic processes (El-Sherbeny and Da Silva, 2013). Aspartic acid treatment is claimed to cause more increases in phenolic contents in our study and confirmed the positive role of different amino acids on increasing phenolic contents of broccoli and flax plants, respectively (Shekari and Javanmardi, 2017). In another study, they linked Asp’s protective role to phenolic compounds derived from amino acids (Heldt and Piechulla, 2010).

Proline is a multifunctional amino acid. It acts as an osmoprotectant and aids in osmotic adjustment, free radical deactivation, nutritional absorption modulation, and CO_2_ assimilation rate induction (Pervaiz et al., 2019). When plants are exposed to harsh climatic circumstances, these functions become even more crucial. As a result, a rise in proline in these plants may have acted as a protective factor, favoring an increase in CO_2_ absorption rate and plant development (Alfosea-Simón et al., 2021). Our results reported that Asp increased endogenous proline concentrations (**Table 2**), consequently improving the plants’ physiological and morphological characteristics (**Table 7**).

Exogenous application of Asp ameliorated the stressful effect of irrigation with saline water in the present research, the increased morphological parameters, including shoot length, leave the area, and shoot dry weight of wheat plant were reported under Asp foliar treatment (**Table 7**). Our results on stress caused due to salinity impact on wheat plants agree with those obtained earlier on sunflower plants (Abd El-Hameid and Sadak, 2020). Salinity stress severely disturbs plant growth *via* various physiological processes (Mujeeb-Kazi et al., 2019; Saddiq et al., 2019; Saddiq et al., 2020). Salinity affected wheat growth parameters *via* decreased water availability and increased levels of Na and Cl ions which interfered with essential macro elements such as Mg, K, and Ca uptake from the soil, thus resulting in nutrient deficiency (Awad et al., 2012; Saddiq et al., 2019; Saddiq et al., 2020; Mohamed et al., 2021). Also, ion toxicity and the osmotic stress caused by high salt concentrations caused an imbalance in crucial metabolic processes due to oxidative stress (Chinnusamy et al., 2006). Previous works confirmed the protective effect of Asp on growth parameters of crops, including rice (Rizwan et al., 2017a), wheat (El-Awadi et al., 2017), and soybean (Sadak et al., 2020). Amino acids play a crucial role in protein metabolism and assimilation. Further, amino acids play a role as stress reduction agents, an essential basis of nitrogen, and hormone antecedents (Maeda and Dudareva, 2012), thus causing the induction of growth traits of crop plants. The application of Asp to wheat plants was found to be effective in enhancing their vegetative development in this experiment.

The present study relied on TT biplot graphs to analyze the impact of employed treatments on the aimed features in one graph (Yan et al., 2000). The first two PCs should reflect more than 60% of the total variation to attain the goodness of fit for the TT biplot model (Yan and Kang, 2002). The current findings summarized and corroborated the results obtained by analysis of variance and mean comparisons (**Tables 1**– **8**). Other researchers have observed similar findings on wheat (Sabaghnia and Janmohammadi, 2014; Rasul et al., 2015).

## 5 Conclusions

To summarize the results of this study, salt stress (salinity) decreased wheat growth characteristics (shoot length, leaf area, and shoot dry weight), along with photosynthetic pigments and endogenous indole acetic acid. NaCl stress reduced total carbohydrates, total flavonoid content, beta carotene, lycopene, and free radical scavenging activity (DPPH%). Aspartic acid treatment, on the other hand, boosted photosynthetic pigments and endogenous indole acetic acid, which improved plant leaf area and, as a result, increased biomass dry weight in both salt-stressed and non-stressed plants. The antioxidant system includes antioxidant enzymes and non-enzymatic antioxidants was upregulated by aspartic acid application, resulting in a reduction in reactive oxygen species accumulation (ROS). Under salt or non-salt stress conditions, reduced ROS in aspartic acid-treated plants resulted in lower hydrogen peroxide, lipid peroxidation (MDA), and aldehyde levels. Furthermore, aspartic acid foliar spray improved compatible solute accumulation (amino acids, proline, total soluble sugar, and carbs) and DPPH% and enzymatic ABTS% radical scavenging activity. The results showed that there was a significant positive association between SDW and each of Chl *a*, Chl *b*, TPP, IAA, TC, TFC, B-Car, Lyco, DPPH, SL, and LA, while significant negative associations were reported between SDW and each of Car, POX, SOD, CAT, NR, H2O2, MDA, and ALD. According to the findings, the quadratic regression model described 100% of the variation in shoot dry weight (SDW) yields. The SDW was projected to increase by 956 mg per plant when the aspartic acid application concentration was increased by 1.0 mM. If aspartic acid is applied at 0.95 mM in the quadratic curve model, the SDW is estimated to be 2.13 g plant^-1^. This study concluded that the exogenous application of aspartic acid mitigated the adverse effect of salt stress damage on wheat plants and provided economic benefits.

## Data availability statement

The datasets presented in this study can be found in online repositories. The names of the repository/repositories and accession number(s) can be found in the article/supplementary material.

## Author contributions

All authors listed have made a substantial, direct, and intellectual contribution to the work, and approved it for publication.

## Funding

The authors extend their appreciation to the Researchers Supporting Project number (RSP-2021/298), King Saud University, Riyadh, Saudi Arabia. This work was part of Research Project No. 11030129 supported by the National Research Centre, Cairo, Egypt.

## Acknowledgments

The authors extend their appreciation to the Researchers Supporting Project number (RSP-2021/298), King Saud University, Riyadh, Saudi Arabia.

## Conflict of interest

The authors declare that the research was conducted in the absence of any commercial or financial relationships that could be construed as a potential conflict of interest.

The handling editor AZ declared a past co-authorship with the author AES.

## Publisher’s note

All claims expressed in this article are solely those of the authors and do not necessarily represent those of their affiliated organizations, or those of the publisher, the editors and the reviewers. Any product that may be evaluated in this article, or claim that may be made by its manufacturer, is not guaranteed or endorsed by the publisher.
